# Bacteremia by non-O1/non-O139 *Vibrio cholerae*: Case description and literature review

**DOI:** 10.7705/biomedica.6716

**Published:** 2023-09-30

**Authors:** José Y. Rodríguez, Carolina Duarte, Gerson J. Rodríguez, Lucy Angeline Montaño, Miguel A. Benítez-Peñuela, Paula Díaz, Olga López, Carlos A. Álvarez-Moreno

**Affiliations:** 1 Grupo de Infectología, Centro de Investigaciones Microbiológicas del Cesar, Valledupar, Colombia Centro de Investigaciones Microbiológicas del Cesar Valledupar Colombia; 2 Departamento de Medicina Interna, Facultad de Medicina, Universidad Cooperativa de Colombia, Santa Marta, Colombia Universidad Cooperativa de Colombia Universidad Cooperativa de Colombia Santa Marta Colombia; 3 Grupo de Microbiología, Clínica Alta Complejidad del Caribe, Valledupar, Colombia Clínica Alta Complejidad del Caribe Valledupar Colombia; 4 Grupo de Microbiología, Instituto Nacional de Salud, Bogotá, D.C., Colombia Instituto Nacional de Salud Bogotá, D.C. Colombia; 5 Programa de Infectología, Facultad de Medicina, Universidad Nacional de Colombia, Bogotá, D.C., Colombia Universidad Nacional de Colombia Universidad Nacional de Colombia Bogotá, D.C. Colombia; 6 Grupo de Infectología, Clínica Universitaria Colombia, Clínica Colsanitas, Bogotá, D.C., Colombia Clínica Universitaria Colombia Bogotá, D.C. Colombia

**Keywords:** *Vibrio cholerae* non-O1, bacteremia, virulence factor, *Vibrio cholerae* no-O1, bacteriemia, factores de virulencia

## Abstract

Bacteremia by non-O1/non-O139 *Vibrio cholerae* is a rare entity associated with high mortality rates. We report a case of non-O1/non-O139 *V. cholerae* bacteremia confirmed by polymerase chain reaction and agglutination tests. The clinicoepidemiological characteristics and therapeutic options for this infection are also described.

## Resumen

La bacteriemia por *Vibrio cholerae* no-O1/no-O139 es una entidad poco frecuente que se asocia con altas tasas de mortalidad. Se reporta un caso de bacteriemia por *V. cholerae* no-O1/no-O139 confirmado por reacción en cadena de la polimerasa y test de aglutinación. Se describen las características clinicoepidemiológicas y las opciones terapéuticas para esta infección.

*Vibrio cholerae* is a gram-negative, facultative anaerobe, halophilic, curved rod-shaped bacterium, ubiquitous in aquatic and estuarine environments. The more than 200 serotypes of *V. cholerae* are distinguished from each other by its surface lipopolysaccharide: the O antigen. The strains belonging to serogroups O1 and O139 are capable of producing cholera toxin (encoded by the *ctxA* and *ctxB* genes) and toxin-co-regulated pilus colonization factor (*TcpA* gene), responsible for secretory diarrhea and intestinal colonization, causing epidemic cholera ^1^.

In the Americas, the most significant cholera outbreak occurred in Haiti. It began in October 2010, affected more than 820,000 people and killed 9,792. In October 2022, the national authorities notified confirmed cases of *V. cholerae* O1 in the greater *Port-au-Prince* area after more than three years without reported cholera cases in Haiti (the last confirmed was in January 2019). By May 2023, 42,351 suspected cases were reported, including 2,678 confirmed cases.

Similarly, by the same date, 99 confirmed cases had been reported in the Dominican Republic ^2^^-^^5^. In Colombia, the last cholera cases were reported in 2004 in Tumaco (Nariño) ^6^. Currently, cholera persists as a public health problem in countries of Asia and Africa ^7^.

Serogroups other than O1 and O139 are called non-O1/non-O139 (NOVC), are responsible for sporadic but significant infections, and are a relatively understudied human pathogen class. They can cause asymptomatic colonization of the gastrointestinal tract ^8^. Some of these strains have additional virulence factors contributing to their pathogenicity and increasing the possibility of invasive infections ^9^.

Acute gastroenteritis is the most common clinical manifestation of non-O1/ non-O139 infections. Infections by these bacteria can affect the biliary tract, skin and soft tissues, and the urinary tract; and can cause bacteremia, peritonitis, pneumonia, and unfrequently, endophthalmitis, intra-abdominal abscesses, meningitis, and external otitis ^10^^-^^12^.

Although non-O1/non-O139 bacteremias are uncommon, they have the highest mortality rate among the infections produced by these serogroups (up to 39%) ^10^^,^^13^. For this reason, the infection’s clinical and epidemiological aspects should be fully elucidated to improve early diagnosis and establish adequate therapeutic strategies ^14^^,^^15^.

## Case description

A 79-year-old woman from the northern coast of Colombia, with a history of arterial hypertension, consulted for an eight-day clinical picture of diffuse colic-type abdominal pain associated with constipation. Initially, the patient was treated with laxatives and enemas, with subsequent pain exacerbation and the appearance of abdominal distension and flatus absence.

**Figure 1:**
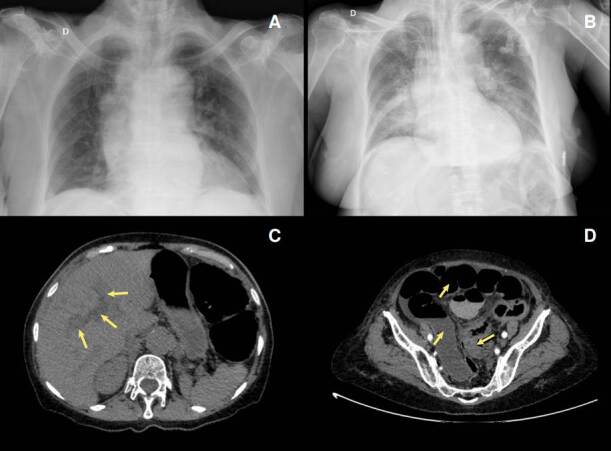
A) Chest X-ray shows cardiomegaly and bilateral hilar lymph node enlargement. B) Chest X-ray, taken 24 hours after the initial test, indicates cardiomegaly and bilateral alveolar infiltrates predominantly on the right side. C) Simple and contrasted abdominal computed tomography showing intrahepatic dilated bile duct (arrow). D) Marked distension of the intestinal loops (arrow), with thickening, showing a decrease in the intestinal lumen at the sigmoid colon (arrow).

Upon admission, in the physical examination, she had a blood pressure of 143/91 mm Hg, a mean arterial pressure of 108 mm Hg, a heart rate of 74 BPM, a respiratory rate of 15 BPM, and temperature of 36.8°C. She presented a distended abdomen without signs of peritoneal irritation and no skin lesions. In the paraclinical findings there were 12,000x109 leukocytes/L, 80% neutrophils, hemoglobin of 9.4 g/dL, 517,000x10^9^ platelets/L, 4.3 mmol/L potassium, 142 mmol/L sodium, 106 mmol/L chlorine, creatinine levels in 1.1 mg/dL, and ureic nitrogen of 43 mg/dL. The initial chest X-ray showed cardiomegaly without alveolar infiltrates (figure 1A).

A clinical picture of intestinal obstruction was considered and managed with hydration and antispasmodics. After five days of hospitalization, the patient presented a compromised consciousness state, an altered respiratory pattern, hypoglucemia, and sustained hypotension.

The paraclinical findings were: 20,300x10^9^ leukocytes/L, 89% neutrophils, hemoglobin of 9.0 g/dL, and 600,000x10^9^ platelets/L. Blood chemistry resulted in 4.4 mmol/L potassium, 145 mmol/L sodium, 105 mmol/L chlorine, 196 U/L aspartate aminotransferase; 80 U/L alanine transaminase; 0.7 mg/ dL total bilirubin, 0.44 mg/dL direct bilirubin, prothrombin time of 10.5 s, partial thromboplastin time of 34.4 s, and arterial-blood gas metabolic acidosis with 7 mmol/L serum lactate.

Chest X-ray revealed multiple bilateral perihilar alveolar infiltrates. Abdomen contrast-enhanced computed tomography showed cholelithiasis, intra- and extrahepatic dilated bile ducts, marked distension of the intestinal loops, and bowel wall thickening with intestinal lumen obliteration at the sigmoid colon level (figure 1B-D). A septic shock of abdominal origin was considered. Blood cultures were taken. The patient initiated invasive ventilatory support and treatment with intravenous injection of 4.5 g piperacillin/tazobactam every six hours, plus 500 mg metronidazole every eight hours. Vasopressor therapy was added with norepinephrine and vasopressin. Persistence of the distended abdomen was associated with voiding of fecaloid material through an orogastric tube. After 24 hours of antibiotic management, the patient presented multiple asystolic episodes without response to resuscitation and died.

**Figure 2:**
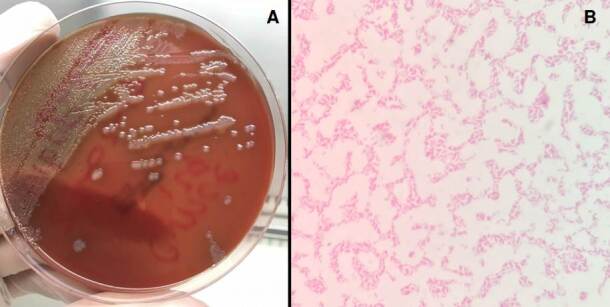
A) The blood agar shows large, smooth hemolytic colonies with uniform edges, surrounded by light areas. B) Gram staining evidencing gram-negative bacilli curved and not sporulated.

Blood cultures were positive for oxidase-positive hemolytic colonies on blood agar, and microscopic analysis revealed Gram-negative curved bacilli (figure 2A-B). The strain was identified as *V. cholerae* by Vitek Compact 2 (BioMérieux, France), Microscan Walkaway (Beckman Coulter, USA), and MALDI-TOF- MS (BioMerieux). The strain was sent to the Colombian *Instituto Nacional de Salud* where *V. cholerae* was confirmed by polymerase chain reaction (PCR), without amplification of the *ctxA* and *TcpA* genes and O1 and O139 serogroups. An agglutination test (polyclonal antiserum O brand) indicated the absence of agglutination with O1 sera ^16^.

A susceptibility profile was performed using the Microscan Walkaway® system. Based on the cutoff points for *V. cholerae*^17^, the strain was sensitive to penicillins, cephalosporins, carbapenems, aminoglycosides, quinolones, and trimethoprim/sulfamethoxazole, with intermediate sensitivity to tetracyclines.

### Patient consent

A patient’s brother provided her written consent. This report does not include elements requiring the approval of the Institutional Review Board.

## Discussion

A hypothesis states that climate change and increased sea surface temperatures, especially during warmer months, favor *V. cholerae* proliferation on phytoplankton and zooplankton, leading to increased concentrations of these microorganisms in filter-feeder shellfish ^18^^,^^19^.

Since 2010, cholera surveillance has been intensified in Colombia ^6^^,^^20^, and 650 presumptive samples of *V. cholerae* have been sent to the *Grupo de Microbiología* of the *Instituto Nacional de Salud*. Out of the 650 samples, 35.2% (n=229) were identified as *V. cholerae* non-O1/non-O139. Nineteen-point-two percent (n=44) were samples from a biological origin (faecal samples and blood cultures, among others), 79.5% (n=182) were environmental samples, and 1.3% (n=3) were food samples (unpublished data, *Grupo de Microbiología*, *Instituto Nacional de Salud*).

Serogroups of *V. cholerae* non-O1/non-O139 have virulence factors that allow them to grow in hyposaline conditions, in addition to genes encoding the regulatory protein ToxR, secretion systems type III and IV (linked to intestinal epithelium colonization), heat-stable enterotoxin, hemagglutinin protease and alpha hemolysins (suggestive of enteroinvasive capacity) ^8^^,^^9^. Many of these virulence factors have been related to some strains causing invasive human infections.

Non-O1/non-O139 bacteremia is rare. Deshayes *et al*. found 350 cases published in the literature between 1974 and 2014. Most cases (45%) originated in Taiwan, 20% in the United States, and 6% in Spain ^1^. We used the same Deshayes’ search criteria in Medline from 2015 to June 2023 and found 30 additional reported cases. This disease predominates in middleaged men (average age of 56 years; male/female rate: 3.3 to 1) and is rare in children under 18 years ^1^^,^^13^.

As in the mentioned case, the most common clinical presentation involves gastrointestinal symptoms (diarrhea, abdominal pain, vomiting, jaundice, and lack of appetite) associated with body temperature alterations (hypothermia or hyperthermia) ^21^. Some patients have lower limb pain associated with inflammation as the first symptom. In some series, hemorrhagic bullae have been described as a risk factor for poor prognosis ^10^. The mortality of this pathology is high and varies between 27 and 39% ^1^^,^^10^^,^^13^.

The bacteremia origin in these patients may be secondary to spread from the small intestine (for example, by an episode of gastroenteritis) or to a skin or soft tissue infection (for example, patients with skin wounds immersed in contaminated water). More than 90% of the patients with bacteremia due to *V. cholerae* non-O1/non-O139 have a predisposing factor. The most common are cirrhosis or other liver diseases (69%), cancer (21%), and diabetes mellitus (13%). Other risk factors identified are alcoholism (16%), biliary tract disease, and steroid use. Patients with cirrhosis are susceptible to bacterial translocation due to inflammation and mucosal edema leading to an intestinal permeability increase, in addition to immunological changes due to alterations in iron metabolism, phagocytosis, and complement, or hepatic reticuloendothelial system bypass secondary to abnormal flow in the portal vein due to portal hypertension.

The infection source is identified in less than 25% of the patients. The most common is raw or undercooked shellfish consumption (54%), followed by contaminated water exposure from marine coasts, lakes, and rivers (30%) and contaminated water ingestion (11%) ^1^. Up to 8% of patients may have skin wounds as a gateway for infection ^10^. In more than 75% of patients, there is no evidence of an identifiable infection source, and one of the explanations may be asymptomatic human and animal carriers with prolonged expression of *V. cholerae* non-O1/non-O139 in fecal matter ^8^.

In this case, there was no clear infection source, and without a history of recent shellfish or fish consumption or fresh or saltwater immersion. However, a limitation of this report is the exposition of a single clinical case and that clinical or epidemiological characterization could not be obtained.

The diagnosis and early initiation of adequate antibiotic therapy can improve the prognosis of patients with non-O1/non-O139 *V. cholerae* bacteremia, considering the absence of guidelines for its treatment ^22^. This microorganism is usually sensitive to β-lactams, tetracyclines, quinolones, and trimethoprim/sulfamethoxazole. Monotherapy is usually used to treat gastroenteritis, while combined therapy is the treatment of choice for bacteremia or sepsis. Based on antibiogram results, clinicians can consider therapeutics with a third-generation cephalosporin with tetracyclines or quinolones. The treatment duration is usually 14 days. However, it should be extended according to the patient’s clinical response and complication development, such as undrained abscesses or meningitis.

Although vaccination is a useful control strategy, vaccines are directed against O1 and O139 strains. People with risk factors should be educated about the danger of coastal water exposure, especially if they have skin wounds and raw shellfish consumption risk.

Although these cases are infrequent, the increasing number of immunosuppressed patients may increase the number of cases. For this reason, it would be advisable to do molecular analyses based on whole-genome sequence data and phylogenetic methods, which will help characterize historical and novel strains, their virulence factors, and their relationship with the environment.

In conclusion, non-O1/non-O139 *V. cholerae* bacteremia should be suspected in patients with risk factors or healthy people with epidemiological exposure and compatible symptoms. If clinically suspected, appropriate antibiotic therapy should be early administered to improve patients’ prognosis with this clinical onset.
